# Seeing the invisible: extracurricular learning processes and learning outcome as experienced by student volunteers accompanying persons in a socially vulnerable situation to healthcare appointments—an ethnographic study

**DOI:** 10.1007/s10459-023-10303-1

**Published:** 2023-12-01

**Authors:** Merete Tonnesen, Gitte Valentin, Thomas Maribo, Anne-Mette Hedeager Momsen

**Affiliations:** 1 DEFACTUM, Evald Krogs Gade 16A, 8000 Aarhus C, Denmark; 2 Present Address: Department of Public Health, Aarhus University, Bartholins Allé 2, 8000 Aarhus, Denmark

**Keywords:** Extracurricular learning, Student volunteer, Inequity, Healthcare attendance, Qualitative study

## Abstract

Becoming a healthcare professional is a complex process, where learning occurs in various ways. This study explores an extracurricular learning approach, called the Social Health Bridge-Building Programme, designed to address health inequities. Student volunteers accompany persons in a socially vulnerable situation to healthcare appointments. Operating outside the realms of health education, the programme intends to provide an alternative road to training healthcare students to become capable of engaging with diverse populations, and reducing barriers to healthcare access. Based on an ethnographic fieldwork, using interviews and participant observation (“walking along”) as methods, the aim of the study was to explore the learning processes and learning outcomes associated with bridge-building, as experienced by students. Our findings show that this extracurricular learning complemented the formal curriculum, and bridged the gap from theoretical knowledge to practice and to real persons, preparing students for their future roles. The particular positioning of walking alongside or sitting beside persons made the invisible visible, enabling student volunteers to *see* the variety of persons in need of bridge-building, ways of living in a socially vulnerable situation, inequity in health, and *see* the persons, beyond initial impression, fostering a deeper understanding and empathy among the students. Learning outcomes included communicational, relational, and observations skills, and a more comprehensive grasp of the healthcare system's complexity. We conclude that a non-governmental organization, independent of the healthcare system, may have found a novel way of providing extracurricular learning about health inequity to students. Demonstrating how the Social Health Bridge-Building Programme complements formal curricula, the concept could be applicable in other settings.

## Background

Health status is determined by the conditions in which people are born, live and age, and the wider set of forces and systems that shape the conditions of everyday life (WHO, 2023). Hence, the drivers of social inequity in health are complex. Though such drivers may belong to realms outside of the healthcare system, the healthcare system plays a crucial role in alleviating the consequences of these social inequities. Ensuring universal access to high-quality healthcare and achieving equitable outcomes, is thus essential to challenging health inequities (Marmot & Allen, 2014). Even in publicly funded healthcare systems, healthcare inequities persist. For instance in Denmark, patients with lower socioeconomic status may experience less treatment compared with other patients (Kjeld et al., 2022).

Becoming a healthcare professional is a complex process, in which learning may occur in various ways. Since healthcare professionals, in the course of their practice, have to collaborate with patients from different backgrounds and carrying different realities, it is essential that healthcare professionals have social competences to interact with all patients. Social competence is here understood as "knowledge, skills, and attitudes that support effective interaction between the physician and patient despite the social distance that separates them", e.g. healthcare professionals being aware of own prejudices and assumptions in social interactions (Loignon et al., 2010, p. 3). Structural competences have also been highlighted as essential, i.e. healthcare professionals' understanding of the social and economic structures that creates and recreates health inequities (Khazanchi et al., 2021; Petty et al., 2017). This includes understanding the complex spider web of structural, communicational, personal, and relational matters that contribute to inequity in healthcare (Kjeld et al., 2022). For instance, the concept of access to healthcare, which is multidimensional, encompasses various structural aspects. These aspects include approachability (making the existence of healthcare services known), acceptability (cultural and social factors influencing whether patients accept services), availability and accommodation, affordability, and appropriateness (the match between patient needs and services) (Levesque et al., 2013). There is also a corresponding set of patient abilities, such as the ability to perceive the need for help, seek help, reach healthcare institutions, pay for services, and engage in healthcare (Levesque et al., 2013). Additionally, the understanding of how access to care may depend on having family or a network to help with access (Tonnesen & Momsen, 2023).

The consequences of no or a limited access to healthcare can lead to a lack of or limited preventive care or a deterioration of health conditions, affecting both the quality of life for patients, and a rise in health costs (Kjeld et al., 2022; Loignon et al., 2015). A first step to change healthcare towards caring for all patients is to understand the mechanisms, complex as they are, that drive inequity in healthcare.

However, social and structural competences among healthcare professionals may be lacking. Studies show that persons in a socially vulnerable situation may feel stigmatized, misunderstood, or not heard in healthcare encounters (Loignon et al., 2015; Ramsay et al., 2019), or that healthcare interventions do not match their needs, or seem too complex (O'Donnell et al., 2016), or there might be a social distance between professionals and patients, living in different social contexts (Loignon et al., 2015).

To improve equitable access for people in a vulnerable situation to quality healthcare that meets their needs and expectations, persons living in a marginalized situation have suggested that professional education should prioritize communication skills, empathy, and understanding of complex lives (O'Donnell et al., 2016).

Clinical experiences among populations in socially vulnerable situations can serve as valuable learning platforms for future professionals. Unwrapping students' processes of learning and the lessons gained from such initiatives can yield important insight to the forming of healthcare professionals regarding their insight to health disparities, and barriers to access to healthcare. Such insights may potentially lead them to advocating and working for greater equity in access to healthcare.

In this article, we explore the extracurricular learning aspects of the Social Health Bridge-Building Programme (from here on bridge-building), an intervention developed to address healthcare inequities by enabling students to co-experience healthcare consultations with patients. Bridge-building differs from for instance internship programmes, as it operates outside the formal educational institutions, through a non-governmental organization, and is not part of students' formal training. Students volunteer, taking non-medical roles, and do not receive supervision from healthcare professionals with regard to bridge-building.

Bridge-building operates in a double temporality: it provides immediate support to persons who otherwise might not attend healthcare consultations, and provides students with experiences that potentially inform their future roles as healthcare professionals (Tonnesen & Momsen, 2023).

We have found no literature on the specific type of volunteering and extracurricular learning that bridge-building entails. However, several studies describe the learning outcomes from student participation in different types of internship among persons in a socially vulnerable situation, or from engaging in and with local communities in healthcare matters. One study describes the learning perspectives of partaking in the community life, for instance how living in remote areas of Australia and engaging in everyday activities helped students contextualize the lives of patients and understand health disparities (Thackrah et al., 2017). Another study on long-term interactions between medical students and patients, accompanying patients to healthcare appointments while taking on non-medical roles, found that it fostered person-centeredness; knowing patients as people contextualized disease with life stories and deepened the students' understanding of the persons' overall situation (Grau Canét-Wittkampf et al., 2020). Experiences of student involvement in clinics targeting persons in a socially vulnerable situation have primarily been examined among North American medical students (Wilson et al., 2023). Their learning outcomes encompass various clinical skills such as interpersonal communication, relational, and collaborative skills, experience in managing language barriers, and awareness of personal strengths and weaknesses, however to ascertain the generalizability of existing findings, more studies from countries with state-funded healthcare systems are needed (Wilson et al., 2023). Other studies have found that a clinical learning experience may result in less fear, and more empathy towards e.g. persons experiencing homelessness (Gardner & Emory, 2018), or cultural competency skills (Asgary et al., 2016; Liu & Li, 2022), and more awareness of their own prejudices, beliefs, and privileges, understanding of needs and social reality, critical perspectives on the health care system, or a rethinking of their vision of good medical practice (Masse et al., 2020; Wilson et al., 2023).

We aim to explore the learning processes and learning outcomes associated with bridge-building, based on students' experiences. Our article contributes to the existing literature on students' involvement with persons in a socially vulnerable situation in two significant ways. First, it includes perspectives from students in various healthcare education programmes (and a few students from theology, anthropology), and from a setting with a state-funded healthcare system. Second, it pertains to a type of student volunteering that is not linked to formal curricula, nor supervised by senior professionals, and students do not perform roles based on their education. It thus provides a unique insight to extracurricular learning.

### Theoretical and conceptual frame

The process of becoming a healthcare professional can involve different curricula, i.e. formal, informal, and hidden curricula. The formal is the actual curriculum at a learning institution, while the informal, or the curriculum-in action (O'Donnell, 2015), encompasses the learning in clinical and non-clinical settings in interactions between students and seniors, creating unscheduled learning moments (Hafferty et al., 2015; Robertson, 2017; Wear & Skillicorn, 2009). The hidden curriculum is the implicit and tacit passing on of "how things work around here" (O'Donnell, 2015, p. 7), i.e. the ideological, or unintended passing on of cultural norms, unfolding between the designed and experienced curricula (Robertson, 2017; Wear & Skillicorn, 2009; Yazdani et al., 2020). Some suggest that the hidden curriculum may lead to *unsee* certain aspects, as medical learning is about shaping the sight on what is absolutely necessary to see (Taylor & Wendland, 2015). Focus in this article is on extracurricular learning, here defined as the exam-free, voluntary based learning that occurs outside formal learning institutions, when student volunteers walk alongside and sit beside persons in a socially vulnerable situation. It is *extra*curricular*,* as it adds extra learning to the formal education, and extra*curricular* as it may not be directly associated with the formal curriculum, yet touches on some of the same aspects that students are expected to learn (e.g. concerning communication, understanding of inequity in health).

We use s*eeing* in our title as an emic term used by informants. However, as indicated by Taylor & Wendland, 2015, *seeing* connects to a wider focus what healthcare students learn to see and how. For instance, the concept of the medical gaze (Foucault, 1973), viewing patients as cases, objectified, separating the body from a person's context, has been defended and criticized: is the medical gaze necessary for medical practice, or a way of dehumanizing medical performance? (Davenport, 2000; Good, 1994). A study on what medical students learn to see in patient encounters in a clinic for homeless persons in the US found that students navigate between gazing and witnessing (Davenport, 2000). Witnessing was an emic term used by clinic volunteers to imply a particular way of attentive listening to the people who go there for help, focusing on a person's life situation, beyond their ailment, and "meeting people where they are" (Davenport, 2000, p. 316). *Seeing,* in our use of the concept, extends the issue of merely observing. It involves taking in a particular situation, using senses beyond sight, to expand one's understanding of situations of social vulnerability. Seeing involves the exposure of aspects of social vulnerability hitherto invisible to the student. We use *seeing* as a lens to understand and to discuss data with.

Finally, a note on vocabulary: In Danish, *ulighed,* usually covers the notions inequality and inequity; neither notions are innocent words but invested with preconceptions and different ways of interpreting them. We here refer to healthcare inequity as those differences which are unfair, unnecessary, and avoidable (EuroHealthNet, 2023). In our title, we use socially vulnerable. While vulnerability is an elastic concept (Sundhedsstyrelsen, 2022), it is also an existential phenomenon that we all live with (Martin, 2021). Vulnerability can be linked to ways and conditions of living, and it can be visible or invisible (Tonnesen & Momsen, 2023). In our use of social vulnerability, it encompasses both a vulnerable socioeconomic status, as the majority of persons using bridge-building have low income and a low employment rate (for those in working age) and relational vulnerability, as family or intimate others in our study seemed to be a limited (or absent) resource among informants who used bridge-building (for elaboration see (Tonnesen & Momsen, 2023). Social support to help attend healthcare appointments was therefore limited.

## Methods and material

This qualitative study is based on an ethnographic fieldwork conducted by the first author, an anthropologist, over a period of 10 months between January 2022 and May 2023.The study is grounded within phenomenology, viewing the body as the entity from and by which humans experience the world, and attending to the intersubjective and temporally informed ways that informants engage in the world (Desjarlais & Throop, 2011). As our aim was to unfold the processes of learning, and the learning outcome, as experienced by student volunteers, an ethnographic fieldwork allowed us to follow such processes closely, attending to the intersubjective way students engaged in bridge-building. Elsewhere, we unfold bridge-building as experienced by different stakeholders (Tonnesen & Momsen, 2023), and describe it through a programme theory (Valentin et al., 2023).

### The social health bridge-building programme

The Social Health Bridge-Building Programme operates in several Danish cities by a non-governmental organization, Social Health. The aim of the programme is to enhance equity in health by enabling healthcare attendance for persons in a socially vulnerable situation, and by educating future healthcare professionals about inequity in access to healthcare. The expectation is that students develop communicational skills and "respectful relations" with persons in a vulnerable situation, and that experiencing the barriers that users may encounter will broaden their understanding of inequity in healthcare (SocialSundhed, 2023). Persons with personal experiences of living in a socially vulnerable situation are active partners in Social Health, e.g. participating in (1) recruitment meetings at educational settings to present their stories, and what Social Health entails, (2) in the Social Health National Board, and (3) in designing course modules for bridge-builders.

The (non-medical) staff the Social Health office has a central coordination role in bridge-building assignments, managing the phone-calls when users, relatives, professionals, and bridge-builders phone to either book an assignment, or to get help if something goes wrong during an assignment, or have questions about an assignment. The office staff briefs the bridge-builder by sending a description of the assignment by encrypted mail, and debriefs after assignment via phone, e.g. how did it go, any obstacles or dilemmas, or need for arranging a new accompanying. Structures of practical and emotional support for bridge-builders include group-based professional supervision. The office staff plans coordination meetings every 6–8 weeks, where bridge-builders meet to plan who is on duty when. The office line is open also outside normal office hours as some assignments take place early evenings. As a principle, Social Health does not reject persons who ask for help, based on an understanding that persons only reach for help because they need help. Terms and conditions of accompaniment are agreed with the individual concerned. Healthcare appointments involve various settings and types of appointments, for instance:

A nursing home professional contacts the office. John, who has dementia and no supportive network that can help him, needs to go to the local hospital. The bridge-builder accompanies John from the nursing home to the hospital. Upon returning, the bridge-builder briefs the staff, in accordance with John. Or, Sandra, living at a half-way house, who asks for help to be accompanied to fetch medicine against drug abuse at a clinic for substance users. The assignment starts and ends at the halfway house.

The persons who use bridge-building are from their 20s and upwards. Almost 4 of 5 persons live on their own. The majority live on state subsidies (including old-age pension).

Of the approximately 200 bridge-builders, the majority are women, primarily students from healthcare educations (public health, medicine, nursing, physiotherapy, occupational therapy, psychology, radiography), and a handful from theology, anthropology, and sociology. Most get acquainted with bridge-building via Social Health recruitment meetings at their place of study or from co-students who are bridge-builders. Students join the programme on voluntary basis; bridge-building is not affiliated with formal educational institutions. Upon volunteering, bridge-builders participate in a 20-h mandatory course and sign a confidentiality agreement.

The course modules are designed by persons with educational background in social work and recovery, some with personal experiences of social vulnerability. The modules contain elements of theoretical, practical (case-based) and experience-based (role-play) knowledge, much alike the forms of knowledge presented by Aristotle (Grøn et al., 2012). Concepts important to Social Health include *recovery* (the understanding that you can recover when suffering from addiction or struggle with psychiatric problems, that a person's self-knowledge is important, and interventions should be done with not for the person), and the related *substitute hope*, explained by one of the designers of the bridge-building modules, "there are good and bad days, there is substitute hope – that's what we are, volunteers who show up, yes, representing hope, for sure".

**Fig. 1.**
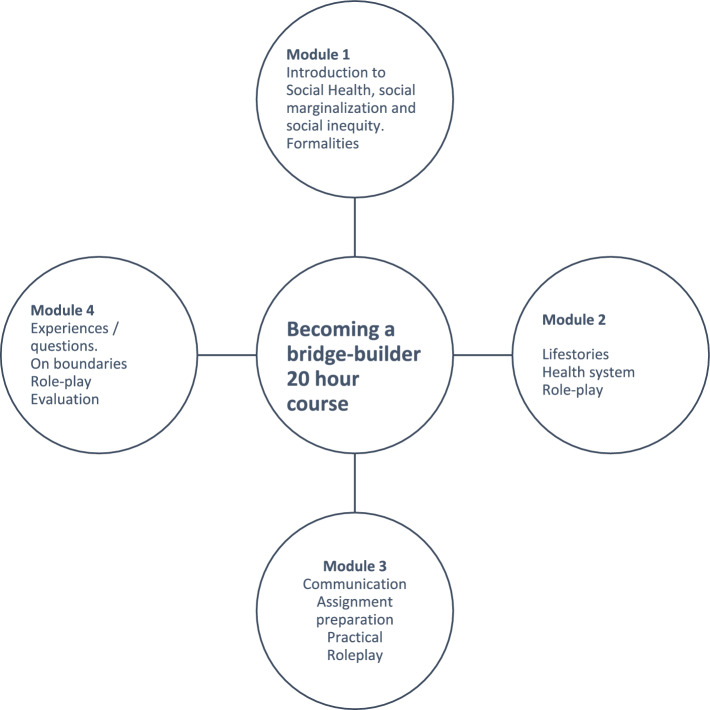
20-h course, four modules

The model (Fig. 1) depicts the modules, followed by a short description.

### Sampling and recruitment

Informants were students from different disciplines (nursing, medicine, public health, psychology, and theology), all active in the programme, with experiences from bridge-building from 4 months to 4 years, and assignments once or twice a month. The fieldwork was conducted in one of the larger cities of Denmark.

To avoid selection bias (i.e. that informants were sampled by Social Health), specific dates for following bridge-building assignments were set, and the students on duty that day were asked by the Social Health office staff if an anthropologist could follow them on assignments. It was emphasized that staff at the office would contact the individuals in need of bridge-building to ask for their permission as well. An informational letter about the study had been distributed to all bridge-builders via the Social Health office. In case of overlapping assignments, the office provided the anthropologist with details about the assignments and the educational background of the bridge-builders, which enabled securing a varied sample of bridge-building assignments and following students from various educations. Students followed during assignments were asked if they would participate in a later interview. A few declined due to practical reasons (e.g. busy with exam). In one case an assignment was cancelled, yet the bridge-builder participated in an interview.

Focus group participants were recruited via Social Health coordination meetings. They had been informed of the focus group beforehand via mail, and told it would take place before the meeting. Participants were bridge-builders attending the meeting. One focus group took place in the city where assignments were followed; another was conducted in another larger city to probe into the generalizability of the findings.

### Methods

Participant observation and interviews were used as primary methods (Hammersley & Atkinson, 2007). Participant observation was chosen to explore how bridge-building is enacted in practice, and to explore the learning process of bridge-building. Before and during participant observation, bridge-builders and users were asked if first author could follow the appointment; users decided the limits of first author attendance. First author participated in 19 bridge-building assignments (equivalent to the approximate number that bridge-builders do in a year), most lasting half a day, spent 6 days at the Social Health office, and participated in three bridge-builder meetings and two general assemblies. In this walking fieldwork (Irving, 2017), as we moved across town using different modes of transportation, "moving time" yielded a space for small talk, and illuminated the different types of assignments, and the interaction between stakeholders. First author positioned as part of the assignment, though discreetly and in a withdrawn way, to limit impact on the situation. I.e. participated in small talk, and helped with practical aspects if necessary, both because the situation was so intimate that it would be awkward not to participate, but also because it illuminated the embodied way of learning, i.e. feeling the anxiety of the person, or handling a wheelchair, helping with a jacket. Being a female, a generation older than bridge-builders, and an experienced fieldworker among persons in a vulnerable situation, first author could relate to some of the situations that might be new to the students, which might have eased access to certain situations. Descriptive fieldnotes were taken straight afterwards, and expanded later the same day or the days after with reflexive fieldnotes (Emerson et al., 1995). Fieldnotes were valuable to remember small-talks, ways of walking, sentiments, but also as a way to e.g. reflect on own positioning as a researcher.

15 semi-structured individual interviews (Kvale, 1997) were conducted to explore students' reflections about the learning process and learning outcome of bridge-building. Informants were affiliated with nursing (1), medicine (5), public health (5), psychology (4), and all but one had been on assignments where first author participated. An interview guide had been drafted while doing observation in the Social Health office, and discussed with Social Health staff, who all had bridge-building experience. The interview-guide with open-ended questions was used as a guide but with room to follow interesting paths in the interviews. Questions focused on the learning aspects (study related, personally) but also why and how they became volunteers, the bridge-building role, examples of the last three assignments, reflections on the concept bridge-building (bridges, gaps attended to, why the need for bridge-building, when it is most meaningful/not meaningful) experiences of health encounters, and their reflections on bridge-building and inequity in health.

The bridge-builder's and first author's shared experience with a bridge-building assignment made it possible to ask about feelings and bodily responses in particular situations. Interviews generally lasted 1–1,5 h, and took place according to students' wishes. Some were conducted straight after the assignment in a quiet part of the hospital with no one around or at a bench outside, other interviews were conducted within a few days of the assignment using cafés, or the university as venues, and two by phone. Compared to interviews with persons using bridge-building (Tonnesen & Momsen, 2023) student interviews exhibited a high level of reflexivity, and elaborate wordings. Generalized statements were explored, "tell me how you experienced this", or "please exemplify" to ensure trustworthiness. Several expressed that it was interesting to reflect from a shared experience, as they usually went on assignments alone.

Two focus group interviews were conducted, facilitated by first author; one in the city where the fieldwork took place with 16 students participating in a Social Health coordination meeting (four students had also participated in individual interviews), another in another city with four student bridge-builders, also in connection with a Social Health meeting. The aim of the focus group discussions was to generate more empirical data, and to probe into some of the results from a preliminary analysis of data from participant observation and interviews. The interview-guide encompassed some of the questions from individual interviews, followed by questions that arose from the preliminary analysis. Some points from the analysis and excerpts from interviews were shared with focus group participants at the end of the interview, with room for discussion. All interviews, individual and focus group, were recorded and transcribed verbatim.

### Analysis

Analysis was formed in an iterative process between coding, discussing themes, and studying literature. The empirical material encompassed individual and focus group interview transcripts and fieldnotes which were read through in full length. Then the material was coded by first author to find chunks of information and 'first order' themes that go across data (e.g. the transcript, "first and foremost it was the thing about helping others that got me started", became a first order theme: *reasons to sign up as bridge-builder*), followed by a second round of coding to refine the thematic codes, i.e. 'second order' themes (e.g. *helping, getting to know the patient population*) (Madden, 2010). Madden calls this approach to data analysis "writing 'out'", arguing that rather than thinking of the analysis as reducing data, analysis adds value, thus conveying a sense of broadening the perspective (Madden, 2010). In our analysis, the empirical material was read through several times, with attention paid to vernacular expressions (e.g. being *present*) (Jackson, 2012), and disparities in the material were important markers (Kvale, 1997). Nvivo was used to manage data. To ensure a transparent approach, themes were critically discussed among authors. To enhance trustworthiness, points from the analysis were presented and discussed with bridge-builders, and representatives from Social Health.

As mentioned, seeing emerged as an emic term, and became a lens to understand data. In the result section, *seeing* is used to structure sections. Inspired by literature on how students learn to see, and what they learn to see, we discuss *seeing* related to witnessing (Davenport, 2000), and the medical gaze (Foucault, 1973). As will be expanded on later, we presented participants at the end of focus group discussions with a few points on *seeing* and *the medical gaze* from the preliminary analysis of fieldwork. This provided bridge-builders with opportunity to reflect on the terms, and consolidated the analytic importance of *seeing*.

We position and discuss the concept of extracurricular learning in relation to other curricula—formal, informal and hidden curricula.

### Ethics

Informants signed letters of consent concerning the use of anonymized data from interviews and observations, the letters included ethical matters such as access to own transcripts and procedure to withdraw from the study. A verbal introduction to this study was supplemented with a written, crafted in plain language. To protect sensitive and potentially identifiable information, we use pseudonyms, provide no information of places of appointments and slur recognizable aspects. See the section "Ethics approval and consent to participate" for more information.

## Results

We present our findings from exploring learning processes and learning outcomes associated with bridge-building, in three parts. The first part concerns becoming a bridge-builder—why and how. The second part focuses on learning processes and ways of seeing: *seeing* the invisible, which links to the inequity and social vulnerability, and *seeing* the person. The third concerns the learning outcomes. We include quotes from a broad range of informants, and indicate, where deemed necessary, the informant's educational background.

### Becoming a bridge-builder: why and how

The majority of the students interviewed became acquainted with bridge-building through an introduction made at their faculty by Social Health, or via other students.

#### Why volunteer

But why did they, young adults in their 20s, beginning 30s, volunteer to do bridge-building? Answers were manifold, but the majority volunteered to *help others* [to a health appointment] and to *gain professional experience* by observing clinical encounters and expanding their knowledge about patient groups, here expressed by two bridge-builders:

[I become a bridge-builder because I thought that] it would be great to be able to help others and at the same time get some experiences with what it's like to be at the other side, as a clinician. Because that's where I will be sometime in the future.

Bridge-building provided a unique chance for someone like me, who comes from a privileged background, a close knit family with no health challenges – it sounded exciting to get a chance to gain knowledge on different patient groups and get a feeling of what it means to be a patient.

The mentioning of "a privileged background" expresses a reflexive awareness of social distance being as aspect in clinical encounters. Another reason was to *make a difference* or *doing something to counter inequity in healthcare.* In this respect, informants often mentioned how, at the introduction to Social Health at their place of study, they were "touched" by the personal accounts of Social Health representatives with personal experiences of living in a social vulnerable situation. As we see in the following quote, incentives for volunteering may shift over time.

In the beginning, it was fighting injustice: This is my city, I live here, and I don’t want it to be like this. Now, as I am about to become a doctor, I [make observations to improve my clinical skills].

Other reasons to volunteer were to *become part of a group*, having moved to a new city, or because it *looks good on a CV*, as several mentioned.

I immediately decided I have to be part if this, I'd like to make a difference. I did voluntary work before and I've always found it rewarding to give something to others. Yes, that's one part, the other is that it looks good on my resume.

Generally, they associated voluntary work with being resourceful, thus anticipating that bridge-building experiences added a signal of resourcefulness to their curriculum vitae. Many had been doing voluntary work before; several argued that volunteering "says something about you", pondering who volunteers and who does not? Their reflections indicated that bridge-builders may have a predisposition for interest in inequity in healthcare and moral standards of interacting with patients. Probing into this in focus group interviews, some informants dryly responded that some of their co-students would definitely *not* volunteer.

Concerning the 20-h course to become a bridge-builder, they generally found that the course provided the necessary information to understand their role as a bridge-builder. Some information was recognizable from their formal curriculum, whereas they found the personal stories of living in a vulnerable situation valuable and "touching". While the role play seemed a bit awkward, most found it educative, "it's a new situation, so practicing for instance what to say when they open the door, how to introduce yourself, was relevant".

#### Why continue volunteering?

Reasons for volunteering gives an indication of incentives for this particular kind of volunteering. However, it may be even more interesting to gain knowledge of reasons for continuing beyond the first few months, as such reflections are embedded in bridge-building experiences. We therefore posed the question: "what makes you continue bridge-building"? While there was a certain overlap to the reasons they volunteered in the first place, they particularly emphasized the inter-human aspect*:*

I still think it's interesting, the human to human encounter, without having work commitments as I would have in a job, I am not the one setting the agenda – it's about being with the person, getting to know them, and try to find out how I can help.

In describing the human to human encounter, they used adjectives as "exciting", "cozy", "just unplug and *be*", "tangible", that assignments made them "feel good", "proud", or "happy" as they were met with gratitude and felt they were making a difference. A very common sentence across interviews was "it takes so little of me and means so much to another person". Several emphasized the quality of presence, deriving a sense of satisfaction from the experience, as this bridge-builder:

I simply love relations and meeting new people – I used to appreciate long, deep relations, but here I find that one hour meetings also provide depth. You are present, not thinking about anything else and it's like [lowers her shoulders] ahhh, now you've done something good, your mood is good and it feels like the sun shines even if it rains.

The issue of a brief meeting providing depth in conversations was a common reflection, and also became evident when walking along assignments as a researcher. Conversations often oscillated between for instance the weather or practicalities and personal accounts of anxiety, dementia, or the sadness of having no family around. Before their first assignments, bridge-builders worried about awkward situations, with long spells of embarrassing silence. However, they all found it easier than expected to communicate and tune in to a person—some talkative, some not, some with little language due to aphasia or a limited Danish vocabulary, in which case body language made out for words. Moving along during assignments, observations clearly exposed how the inter-human encounter often generated an understanding of having something in common—music, enjoying a cup of coffee, or living in the same city. More so, as several emphasized, when accompanying the same person multiple times, they just picked up the conversation from last time.

### Ways of seeing

Walking alongside and sitting beside in assignments during fieldwork, it became evident how senses were activated in bridge-building. Whether it was the scent of cigarettes lingering in a home, accompanied by the sounds and attentive listening to words, or the taste of a biscuit shared after an appointment, or the comforting touch on a shoulder at the dentist's, all these experiences highlighted the embodied nature of performing bridge-building. However, it was the sense of sight that bridge-builders themselves emphasized. They recognized that there were various ways of seeing, each relating to different scales: an individual's life context, the interpersonal dynamics during healthcare appointments, and the structural aspects of the social system and the healthcare system. Seeing exposed what had prior been invisible to the student volunteers.

#### Seeing the invisible–inequity and social vulnerability

There are so many ways of being a human being and being challenged in life that I never knew.

This line wraps up the essence of many interviews, as bridge-builders emphasized the variety of persons they accompany – "they are all different" was a common expression, often supplemented by examples from different assignments. Indeed, walking along on assignments, examples of differences come to life: an elderly, frail woman accompanied to an eye specialist—the narrow corridors making it difficult to navigate her wheelchair, on the bus ride to a psychologist small-talking with a young man who suffers from anxiety, holding hands of a young woman with a drug abuse at the dentist, coming back with a woman to the halfway house, where she stays, to find a handwritten note on the front door "ring the bell -the door is locked for safety reasons", waiting with an elderly man for a scan at the hospital that might show "why my head is not working. It simply stopped working", sitting beside another as a doctor explains the course of a dementia screening, or waiting for transport home after a cardiac examination with a woman, a refugee, who only knows a few Danish phrases.

The starting line conveys an understanding that bridge-builders often enter spaces that are unknown or invisible to them, thus *seeing* ways of living or "*seeing* people in society that you don't see—like drug users, persons with dementia and so on", as one bridge-builder explained, while another said:

Coming into a home that's a total mess, where nothing has an own set place and think about that this works for this person, you think about the reasons for the home to look like this – so you really meet a broad variety of people… and well, it's not just their personality, their looks, it's also their whole life - their acquaintances, their housing, their home – it's everything! It's a life almost unimaginable, actually, because it is so different from your own background.

Their experiences thus put a lens to their own life, realizing the social distance from their life to lives of those they accompany. Others expressed surprise when realizing or *seeing* the scale of persons who find it challenging to access healthcare:

In my family, we handle healthcare appointments ourselves or help each other if needed. Now, I've seen and experienced and reflected: okay, more people than I had expected actually need this help. I wouldn't be able to point out, just by looking at people in the street, who needs this help. In the beginning I thought, and I know this sounds prejudiced, but alcoholics and persons with psychiatric needs and maybe older immobile persons – but there is so much more in between.

During interviews, a dichotomy materialized between what is visible and what is not. Living in a socially vulnerable situation may be visible, like a man, during fieldwork, his hair unkempt, and with few teeth left, whose pants kept falling down when we walked to the dentist, or it can be invisible, at least at first sight. Several bridge-builders noticed how they had been on assignments where it was not obvious from the beginning why the person needed help, but as many assignments last half a day, they would start noticing small signs. One wondered why a particular person needed accompanying, until the person revealed that had she been alone, she would always look over her shoulder, due to anxiety, "so the invisible was invisible because I was present, whereas it might have been visible had she been on her own".

Indeed, *seeing* exposed how the road (or access) to a healthcare appointment can be full of obstacles: anxiety, problems with wayfinding, physical barriers such as steep steps, or small room with little space for a wheelchair, or not having a reliable network to help making it to healthcare appointments (for elaboration on this point, see (Tonnesen & Momsen, 2023; Valentin et al., 2023):

I'm surprised at the variety of challenges to making it to a healthcare appointment and meeting the healthcare system - language wise, remembering to lock the door, the road to the appointment, that so many people find it challenging to go to the doctor. I think there are multiple small barriers but what's applicable to many of the situations is that it just seems so unmanageable.

Listing barriers, a student found bridge-building a tangible and practical solution, mentioning how many would not make it across the doorstep if not accompanied. She added, "Equality and equity in health is not just a healthcare system that's available to all—some need help to access health. Equity is that you adapt possibilities to the person's resources—I think we need more focus on that". Indeed, most agreed with her, and commended Social Health's principle that if a person asks for bridge-building, they do get help. Most believed that a person only asks for help if needed, and applauded the flexibility: that here, a person does not have to fit into a specific category or be ill enough to get help.

Finally, *seeing* could also pertain to structural issues; several bridge-builders expressed surprise at the inequity in the Danish system, captured well in this quote:

It surprised me just how much inequity there is. I always thought that since it doesn't cost anything to go to the doctor, then everybody can do it, surely. But no. It's not that simple. Even if everybody has the opportunity – well some don't. If your anxiety inhibits you from taking the bus, then you cannot go to the see the doctor.

She recalls a person with multiple health problems AND an itchy skin, who asked the doctor to estimate the price of the recommended lotion. "We're not talking about millions here", the bridge-builder had thought to herself, only to realize that living on a tight budget, allocating money for medicine could be difficult. She used to link inequity in health to for instance the US, where good treatment requires a health insurance, "That's not how it is here, I thought—we can go to the same hospitals and get the same treatment. But then that's actually not right! We need to look at what is hidden". I interviewed her at the university. She looked around at the students engaged in discussions over cups of café latte, then said that actually she was not into politics, but this experience ignited a sense of anger within her. Other bridge-builders also shared how going on assignments spurred a social indignation within them:

[…] like a woman, who suffered terribly from anxiety, I had assignments with her before and after that Indian summer a couple of years ago – when I came back after the summer, I realized I was the only one who had been to see her. So during that hot summer time she had been sitting inside smoking cigarettes. I'm not sure that it's inequity in health as such that surprises me, but rather the awful lives that some people live – just because it is not visible does not mean that it is not there. Well, I have SEEN it.

The quote exemplifies how bridge-builders make use of their senses during their assignments, here seeing, and smelling the cigarettes. This bridge-builder had "SEEN it", which seemed to spur a sense of indignation in him as he continued: "I don't want it to be like this [persons living lousy lives with no network]. So inequity has definitely become part of my view of the city". The statement that nobody else had been to see her touched on another common reflection about *seeing* lives almost devoid of family or network.

To recap, bridge-building made the invisible visible, i.e. the variety of persons in need of bridge-building, life situations, and concrete examples of inequity in health.

#### Seeing the person

*Seeing* exposed inequity or very difficult situations that people live in. However, visibility seemed to expand to seeing behind the first hand appearance, i.e. *seeing* the person, and getting a peep into the life world and life history of the person:

I have had some wild conversations with some of the persons I have accompanied […] telling me openly and honestly about their experiences, which I find SO interesting […] afterwards, I have thought how amazing this willingness to share stories with me is, considering that they don't really know me. I think that's brave.

Moving along conveyed much information about a person's life. The concept, *wild conversations,* captures the surprise expressed by bridge-builders of the depth of conversations and the intimate details of life shared by the persons they accompanied. Some used the word *de-mystify* to express accompanying persons who on first appearance did not resemble "someone you'd walk down a dark alley with", only to find that they had lived "interesting lives that can teach you something", as a student explained. Seeing meant unwrapping and contextualizing a person's life situation:

I kind of thought I'd be less understanding…when you're with a person who may have made choices in life, that are difficult to understand… but when you see this person as a whole, then you realize that there may be aspects beyond this person's powers to change. It's not his or her fault it's just as much the social environment, like drawing a blank.

*Seeing the person* seemed motivated by walking alongside, small talking, often finding a common third, i.e. a shared topic to talk about, or being invited to the home, or waiting together – doing together. This was repeatedly confirmed during fieldwork. For instance, the man whose pants kept falling down. As we waited for his dentist appointment, the bridge-builder's casual comment on an exam coming up, made him open up and share stories of how he used to teach, had next to no family, but did see a former girlfriend now and again, and just before he went to the dentist's chair, he shared the story of him getting the seizure that shook his brain.

Users and bridge-builders have a common goal – to get to and from a health appointment. Bridge-building means moving across town via bus, car, tramway, bicycle or walking. Some bridge-builders commented on experiencing their city anew, now knowing that inside that house with a wild garden in front and paint peeling off the front door lives a man with dementia and a dwindling network besides a niece; he used to love travelling and his living-room is full of ornaments from his travels. Or that this apartment block houses a half-way house for active drug addicts. The city not only became a common room, but also a very concrete and easy to chat about topic of conversation.

Several bridge-builders mentioned creating a certain bond with the person:

Though I'm not the one with a doctor's appointment, it sometimes feels like it. Like I was with a woman to get results from tests and scans. Luckily the news was good and I just felt that it also made me quite relieved. Maybe it matters that we are at the same level, it's not, 'now, I am here to help you', but rather we have an appointment together so it becomes… me being just a regular person [she points to her heart], not any authority.

Seeing the invisible, seeing the person, generated a contextualizing of lives lived, it touched the bridge-builders emotionally, and made them consider their own and others' prejudices. S*eeing* paved way to questioning labels such as "vulnerable", and "marginalized", are such labels befitting for the persons they *see*? As a bridge-builder said: "It could be any of us—you never know what life brings".

### To gain clinical skills

Standing beside them rather than sitting across from them – well that's truly a different role.

This statement came from a student, comparing his experiences of walking alongside to practicing medicine in a clinic as a trainee under supervision of seniors. In this section, we unwrap the learning outcome of this different positioning (walking alongside/sitting beside), which bridge-builders specifically emphasized in relation to their field of study and their future positions. Several common denominators prevailed in their reflections.

#### Meeting the clientele, cultivating knowledge about "the system"

One was a general perception that meeting the clientele was useful for their understanding and knowledge about the persons they would meet in their future jobs:

Somehow it's like [I live in] a bubble. I seldom meet persons such as the ones I accompany, well I didn't before, which is actually a paradox when you think about it – me becoming a psychologist, going to meet people in my future working life. Our clinical practice during my study is limited, but I feel I owe it to the persons that I will be helping in the future - that I have actually been out there, making eye contact.

Students of medicine, psychology, and nursing expected face-to-face interactions with patients in their future positions and generally found bridge-building an add-on to their practical experience of clinical work inherent to their studies. Students of public health, on the other hand, expected little patient contact in their future jobs. They emphasized that inequity in health was a topic in their formal curriculum and how words on paper and statistics came to life during bridge-building, offering concrete examples they could use in class room discussions:

[We] get hands-on experience with persons from marginalized positions, […] it's very theoretical at the university, you show up, there is a lecture, you go home, whereas here, you get a bodily feeling of what it's like to be in the real life and work with real people - bridge-building provides you with other tools [...] those I meet are the ones I'll develop health intervention for in the future. While it may be unhealthy to smoke and have a coke for breakfast, you get another understanding of why it might be so difficult to make the healthy choice.

This student clearly demarcated the difference between marginalized lives and the everyday life as she knows it, and the real life and the university life, a demarcation that materialized in many forms during interviews and in small-talk during assignments. Bridge-building operates in the borderland between these spheres, and according to this quote, seems to bind the spheres together. She also demarcates a contrast between making healthy choices or not, finding that navigating in the borderland broadens her horizon of understanding the choices made by different people.

Another common denominator was cultivating understanding about the complex system. Walking alongside exposed the landscape that patients traverse in the social system and healthcare system. While bridge-builders generally found it illuminating to get acquainted with half way houses, a social dentist clinic, or the queuing system for blood tests, many commented on the complexity of the system. "The healthcare system works well, I think, if you have the resources to navigate it and be in it", as a bridge-builder said, finding the system bureaucratic. Others found that ways of doing things were made for the system rather than the patients, for instance when patients had to go to the hospital several times within a short period of time, instead of "pooling" examinations. Furthermore, that there could be a mishmash regarding who is responsible for what, making some patients "fall between two chairs", i.e. not fitting into the right "boxes" of the compartmentalized health system. As a bridge-builder said, "We generally say that the system is for all, but that's actually not the case because there is only *one* way of using it".

However, the few bridge-builders who lived with chronic illnesses (themselves or close relatives) made the point that they too had experienced how difficult the system can be to navigate when you are not well, emphasizing how difficult it must be if one's network is not resourceful.

#### Observational and communicational skills

Regarding a third common denominator, students expressed a dual perspective concerning observational and communicational skills: they observed what professionals seem to see and do, *and* practised their own observational skills or own gaze; they observed professionals' communicational skills *and* practiced their own communicational skills. Here an example of practising own skills:

An important aspect of being a psychologist is to try to understand how other people feel and what you can do for them. And that's something I do all the time when on bridge-building assignments. It's very much about reading their body-language, their way of talking, their needs. Trying to understand while at the same time being present.

Others observed healthcare professional/patient interactions, finding the learning cross-disciplinary, i.e. coming doctors could learn from e.g. nurses, dentists. Acknowledging the scarcity of time during appointments, some observed the strategies employed by professionals to navigate the conversation to focus on pertinent issues, while maintaining a positive approach:

I learn from assignments – in the future it will be me, sitting alone at the opposite side of the desk, having to remember how patients may have struggled with transport, waited two hours, already anxious about being here, feeling emotional, getting even more information to remember and then wait a couple of hours for transport home. I think it'll be conducive for my future job – tuning in to these patients making them listen to what I say […] I think you ought to experience this as a student of medicine, trying to see things from the other side, how doctors talk to patients and so on.

Tuning in appeared an embodied way of adapting to the pace of the person, noticing discomfort, establishing eye contact and pay attention to small winks and gestures, and seemed to signify a way of reaching or establishing a mutual understanding, and added to bridge-builders' relational skills. A student of psychology found that her experiences left her with "a bank full of intuitions; my body learning how to react in certain situations".

Bridge-builders from public health observed other aspects:

Since I'm not studying medicine, it's not the doctor's role as such that I observe and really take in. Rather, it's how the system is constructed and why it's difficult to make it to a health appointment. How an interpreter may be expected, but does not show up, as once happened in one of my assignments – it's more on a structural, systemic level that I question how viable it all is. To get hold of the doctor you have to phone within this hour, you may get an answer from the doctor but need to get hold of your sister to get her help to understand it – things easily turn complex, right!

Two observations pervaded reflections on communicational skills. One, that time matters—and often seems compressed at the doctor—with little time to tune in, to address the patient's life situation, or provide context to the patient's expressed words. This juxtaposed the rhythm of accompanying which could seem slow in comparison. Students unanimously agreed that time spent with the doctor holds immense value to patients. A medical student shared his experience of a six-hour appointment, with only "about three minutes with the doctor", while another student sometimes thought to herself "you [the doctor] truly have no idea what this patient has gone through to come see you". Two, a tendency for some healthcare professionals to address them, rather than the patient.

Bridge-builders here expressed ethical considerations about their role—should they keep quiet, or speak out in case they felt the professional did not take the time to listen, or they thought the patient did not understand what was being said?

Indeed, micro-ethical considerations abounded in interviews: how to leave a person, when knowing he will be alone for some days, how to politely decline money for the services offered [which also happened to first author], how to make healthcare professionals divert attention to the patient, how to say goodbye after many hours together—by hug, smile, handshake?

#### Expanding the medical gaze

Students from nursing, medicine and psychology emphasized how walking alongside added to their knowledge about diagnoses. One was surprised to learn about new (to her) facets of schizophrenia from a person she accompanied, another had followed a patient's dementia assessment, learning about the different steps to a dementia diagnosis. Another found that no reading about chronic obstructive pulmonary disease could prepare him the way accompanying could, to understand how pervasive and shortness of breath is to everyday life. Others became more attentive to what happens before and after consultations, i.e. how strenuous health attendance can be, or to a person's context, "how people struggle with household chores, economy, social institutions, having to coordinate it all *and* follow a treatment plan at the hospital". Here, patients became teachers, passing on tacit knowledge.

Several medical students believed that learning about the healthcare system from another perspective, formed them as a professional, but also made them realize what to avoid:

I've met several doctors I will definitely NOT become like. As a bridge-builder, you quickly realize if a patient is anxious, but I think that many doctors do not notice this. It's rather- 'okay, we need to check this lump on your head', and then they move on. But recognizing the anxiety and working around it, I believe, will improve my skills as a doctor.

At the end of a focus group discussion, a few points from the preliminary analysis of fieldwork data was briefly introduced. A group of medical students, all at the end of their studies, picked up the concept of "the medical gaze". Someone said, "that's so true, it's actually crazy how fast you gain that medical gaze—in that sense, bridge-building makes an important counterbalance". They all nodded in agreement. Another medical student would probably have agreed. In an interview, he referred to having heard from doctors that, "you might forget to see the person, trying to imagine their life. But it's important to look beyond the result of a blood test, and see: what kind of person is here, in front of me?", he added.

Hidden in the medical students' reflections was a wish to become a doctor who is present, who listens, who knows how to communicate in a respectful manner, including paying attention to the patient, and who is observant to the context of the patient.

To wrap up this section of skills gained, all bridge-builders emphasized that they gained different kinds of knowledge from accompanying persons in a socially vulnerable situation. This extra-curricular learning added to their formal curriculum. It translated words from study books to practice, and to real persons, it transformed life from a "bubble" to exposure to a wider range of ways of living, and it provided contextualization to lives lived, which seemed to create a deeper understanding and empathy among the students. Also, it generated learning about self, e.g. being more understanding and less prejudiced, acknowledging how their own "privileged" background could influence their perception of a given situation, or to remain composed in challenging situations, and engage in dialogues with persons they had just met.

## Discussion

In the Social Health Bridge-Building Programme, student volunteers accompany persons in a socially vulnerable situation to health appointments. Our aim was to explore the learning processes and learning outcomes associated with bridge-building, as experienced by students.

We found that the motivation for volunteering as a bridge-builder predominantly revolves around a desire to help and to gain clinical skills. Additionally, motivation is driven by a commitment to make a difference or address health disparities, or expecting volunteering to improve a CV, others joined to become part of a group. These motivations are not surprising and are largely consistent with other studies on volunteering healthcare students' motivation (Rovers et al., 2016). However, we went one step further, asking why continue volunteering, as this may be a more viable indicator for interest in volunteering. Here, the emphasis was on the human-to-human encounter, which students found interesting and rewarding. They emphasized the quality of presence and how they derived a sense of satisfaction from bridge-building. A common remark was that it took little effort on their part, but mattered greatly to the persons they accompanied.

Bridge-builders are in a betwixt or a liminal situation in the sense that they are not-yet healthcare professionals but in a state of becoming, and because of this, they are neither "just" community members nor fully part of the professional healthcare establishment. Compared to other volunteer programmes for healthcare students, this programme is extracurricular, students are affiliated with various disciplines, they are not supervised by senior healthcare professionals, nor is it a formal skill test situation at a clinical setting. What, in this liminal position, do students learn from bridge-building experiences?

We suggest that learning processes and outcome are closely linked to the positioning of the bridge-builders. They walk alongside, they sit beside. This positioning contrasts learnings through formal and informal curricula, as they do not side with professionals but with the patient. Walking alongside, they sometimes have to deal with unexpected situations, and several bridge-builders refer to such situations as learning process, which in turn makes them more capable as health professionals.

We found that *seeing* was an essential component of bridge-building. Bridge-builders made use of senses and intuition during assignments, but highlighted the sense of sight. The emic term, *seeing,* entailed aspects beyond merely observing. *Seeing* meant taking a particular situation in, expanding one's understanding of the situation. *Seeing* exposed what had been invisible to the student volunteers. Walking alongside and sitting beside opened for ways of seeing: *seeing* the invisible, i.e. homes and lives that do not resemble anything they have seen before; *seeing* just how differently lives can be lived and how social vulnerability has diverse outlooks; *seeing* the signs of inequity in healthcare, finding them unfair, and the scale of persons in need of accompanying, also exposing how family was not always a resource to be counted on for help.

*Seeing* meant unwrapping aspects of a person's life situation that expanded bridge-builders' views of what it means to be in a socially vulnerable situation. They juxtaposed this exposure to their normal "bubble" of a privileged university life, an exposure that to some became a realization of their own preconceptions or prejudices, making a social distance apparent. However, walking alongside also enabled them to *see* the person, and contextualize the life and circumstances of that person. *Seeing* was scalar and pertained to not only an individual's life context, but also seeing behind the appearance of the individual, to the whole person, to the interpersonal dynamics during healthcare appointments, and to more systemic aspects of the social and healthcare system. For instance, a student defined equity as adapting possibilities to the person's resources, which somehow countered other students' descriptions of "only one way of using the system".

We found that learning outcomes included becoming familiar with the clientele of their future professions, gaining observational and communicational skills, and getting an understanding of the complexity of the system, but also acquiring knowledge about concrete places of healthcare.

Those expecting patient interaction in their future jobs viewed bridge-building as a test course for the future, or as several referred to, "observing clinical work from the other side of the table", meaning that bridge-building situations provide an insight into how clinicians work (how they communicate, how they deal with frustration), and also how the system works. To medical students, volunteering as bridge-builder formed, in the words of students, a "counterbalance" to the medical gaze (Foucault, 1973) (for a similar finding, see Davenport, 2000), as they gained more in depth knowledge of how diagnoses manifest in real lives, and realizing how essential it is to contextualize the life situation of a patient. They also got an idea of the kind of professional they would prefer to become, one that could be present, and one that would pay attention to the context of the patient—both in terms of what happened before and after the consultation, and the life situation.

*Seeing* bears a certain resemblance to witnessing, if we use the distinction between gazing (focusing strictly on a body component) and witnessing (focusing on a person's life situation, beyond a patient's ailment) (Davenport, 2000). Witnessing has a moral connotation, which in our study became apparent, when students expressed social indignation—of persons living with no network around them, of the injustice of eschewed access to healthcare even in a country with universal healthcare. Witnessing, or seeing, put a stain on the geographical map of the city, in the form of realizing the inequity present. On the other hand, seeing also enriched students with knowledge about places and ways of performing health activities, and provided nuances to perceptions of healthcare.

Generally, they found that bridge-building experiences made them more open-minded and adept to navigate different situations.

We argue that the Social Health Bridge-Building Programme leads to extracurricular learning, i.e. learning occurring outside formal education programmes. If the hidden curriculum is about the implicit and tacit passing on of norms and how things are done or expected to be done, then the hidden curriculum may lead to see what is not explicitly taught through the formal curriculum. It may also lead to *unsee* certain aspects, as medical learning is about shaping the sight on what is absolutely necessary to see (Taylor & Wendland, 2015). We suggest that the extra-curriculum may lead to *see* well beyond the medical gaze, and further suggest that seeing is grounded in being present with an individual in situations that extends healthcare situations. There may be informal learning embedded in bridge-building, given the observation of professional/patient interactions, but perhaps more essentially, we argue, students learn from situations and from persons, through life-stories and *wild conversations*, through small-talk, by being welcomed into homes and trusted to accompany.

A study found that time spent with the patient seems a prerequisite for learning (Grau Canét-Wittkampf et al., 2020). However, we found that a short, often intense time together could also create valuable learning, even bonding with persons. While we argue that positioning matters, we also acknowledge that learning outcomes are similar to those found in clinical settings (Davenport, 2000; Gardner & Emory, 2018; Grau Canét-Wittkampf et al., 2020; Liu & Li, 2022; Masse et al., 2020; Thackrah et al., 2017; Wilson et al., 2023). So bridge-building seemingly becomes an additional road to learning. An extracurricular learning that may provide more in-depth perspectives as students *see* homes, experience the obstacles to and from a healthcare appointment, and observe interactions from a privileged position, a liminal position, in an exam free space.

Based on our findings, the Social Health Bridge-Building Programme provides a learning platform that do seem to educate future healthcare professionals by broadening their understanding of ways of living, of health communication, and of inequity, i.e. building social and structural competences (Khazanchi et al., 2021; Loignon et al., 2010; Petty et al., 2017). In the liminal position of not-yet being a healthcare professional, but in the state of becoming one, the forming of students entails a mix of formal, informal, and hidden curricula, and, with bridge-building, an extracurriculum where learning takes place by co-experiencing healthcare appointments—sit beside the person, and walking alongside. It could be argued that bridge-building represents a way of practicing distributed health (Edwards et al., 2013), i.e. that patients draw on social network (here bridge-builders) to support them in accessing health information and understanding. In the bridge-building programme it may, however, be more appropriate to understand distributed health in a reciprocal way: students distribute or share their health competences with patients, and patients share their competences and knowledge with students.

Considering that those volunteering may represent a small group with a possible predisposition for interest in health inequity, we do not know whether the Social Health Bridge-Building Programme and the insights gained by the students have lasting effect in their future positions, and whether it has an effect on disparities in healthcare. Given the prospects of a possible new way of extracurricular learning, this deserves exploration in future studies.

### Strengths and limitations

The strength of this study lies in its in-depth explorations through participant observation and interviews, as well as its inclusion of perspectives from various disciplines. But there are some limitations, such as the potential variation in learning outcomes between students with different levels of direct patient contact. While we do indicate distinctive features of learning outcomes among students of medicine and among students of public health, future research should consider in-depth discipline-specific analyses and longitudinal studies, to further explore the differences and explore how the learning outcomes that students imagine they will lean on in future positions, actually come to use. Furthermore, the students may be a homogenous group with a predisposition for interest in inequity in health, which might explain a certain glossy way of referring to health volunteering, as also noted in global health volunteering (Wendland et al., 2016). Finally, it is worth looking deeper into the challenges of ethnicity and language barriers in bridge-building. Some observations involved persons with a limited Danish vocabulary, and in two instances, bridge-builders from different ethnic backgrounds than Danish, unfortunately these students were unable to participate in interviews due to their busy exam schedule.

## Conclusion

This article gives an insight into how a non-governmental organization, with no formal affiliation to the healthcare system, may have found a novel way for students to learn about and contextualize the many shades of social vulnerability and examples of inequity in health. In this unique context, the Social Health Bridge-Building Programme offers new perspectives on how extracurricular actors and activities can contribute to students' learning, including patients becoming teachers. The study also adds new perspectives to existing findings on healthcare student involvement with persons in a vulnerable situation: firstly, our findings stem from a country with a state-funded healthcare system, secondly, bridge-building incorporates students from various disciplines, including those who will have patient contact and those who will work with the structures and the design of healthcare, and thirdly, bridge-building exemplifies learning processes taking place with students volunteering outside formal education settings. The extracurricular learning aspects of bridge-building could find relevance in various global contexts.

## Data Availability

To honour the anonymity promised the study participants, we disclose no data.
